# Lurbinectedin in patients with pretreated endometrial cancer: results from a phase 2 basket clinical trial and exploratory translational study

**DOI:** 10.1007/s10637-023-01383-2

**Published:** 2023-08-09

**Authors:** Rebecca Kristeleit, Alexandra Leary, Jean Pierre Delord, Victor Moreno, Ana Oaknin, Daniel Castellano, Geoffrey I. Shappiro, Cristian Fernández, Carmen Kahatt, Vicente Alfaro, Mariano Siguero, Daniel Rueda, Ali Zeaiter, Ahmad Awada, Ana Santaballa, Khalil Zaman, Jalid Sehouli, Vivek Subbiah

**Affiliations:** 1 University College London Cancer Institute, NIHR UCLH Clinical Research Facility and Guy’s and St Thomas’ NHS Foundation Trust, London, UK; 2 Institut Gustave Roussy, Villejuif, France; 3 Institut Claudius Regaud, Toulousse, France; 4 START Madrid-FJD, Hospital Fundación Jiménez Díaz, Madrid, Spain; 5 Gynecologic Cancer Programme; Vall d’Hebrón Institute of Oncology (VHIO), Hospital Universitari Vall D’Hebrón, Barcelona, Spain; 6 Hospital Universitario 12 de Octubre, Madrid, Spain; 7 Dana-Farber Cancer Institute, Boston, MA USA; 8 PharmaMar, Colmenar Viejo, Spain; 9 Institut Jules Bordet, HUB, Université Libre De Bruxelles, Brussels, Belgium; 10 Hospital La Fe, Valencia, Spain; 11 University Hospital CHUV, Lausanne, Switzerland; 12 Charité – Universitätsmedizin Berlin, Berlin, Germany; 13 The University of Texas MD Anderson Cancer Center, Houston, USA; 14 Present Address: Sarah Cannon Research Institute, 1100 Dr. Martin L. King Jr. Blvd., Suite 800, Nashville, TN 37203 USA

**Keywords:** Lurbinectedin, Endometrial cancer, Phase 2

## Abstract

Second-line treatment of endometrial cancer is an unmet medical need. Lurbinectedin showed promising antitumor activity in a phase I study in combination with doxorubicin in advanced endometrial cancer. This phase 2 Basket trial evaluated lurbinectedin 3.2 mg/m^2^ 1-h intravenous infusion every 3 weeks in a cohort of 73 patients with pretreated endometrial cancer. The primary endpoint was overall response rate (ORR) according to RECIST v1.1. Secondary endpoints included duration of response (DoR), progression-free survival (PFS), overall survival (OS), safety and an exploratory translational study. Confirmed complete (CR) and partial response (PR) was reported in two and six patients, respectively (ORR = 11.3%; 95%CI, 5.0–21.0%). Median DoR was 9.2 months (95%CI, 3.4–18.0 months), median PFS was 2.6 months (95%CI, 1.4–4.0 months) and median OS was 9.3 months (95%CI, 6.1–12.8 months). Molecular subtypes showed differences in PFS rate at 6 months (p53abn 23.7% *vs.* “No Specific Molecular Profile” [NSMP] 42.9%) and median OS (p53abn 6.6 months *vs.* NSMP 16.1 months). The most common treatment-related adverse events (mostly grade 1/2) were fatigue (54.8% of patients), nausea (50.7%), vomiting (26.0%) decreased appetite (17.8%). and constipation, (19.2%). The most common grade 3/4 toxicity was neutropenia (43.8%; grade 4, 19.2%; febrile neutropenia, 4.1%). In conclusion, considering the exploratory aim of this trial and the hints of antitumor activity observed together with a predictable and manageable safety profile, further biomarker-based development of lurbinectedin is recommended in this indication in combination with other agents. Clinicaltrials.gov identifier: NCT02454972.

**Supplementary Information:**

The online version contains supplementary material available at 10.1007/s10637-023-01383-2.

## Introduction

Endometrial cancer is the sixth most common cause of cancer in females with 417,000 cases every year [1]. Patients who progress beyond first-line chemotherapy have a poor prognosis and novel therapy options are urgently needed. The Cancer Genome Atlas (TCGA) study of endometrial cancer identified four molecular subtypes [2]. Based on this, the Proactive Molecular Risk Classifier for Endometrial Cancer (ProMisE) Algorithm has been developed to assess endometrial cancer samples and classifies them in four molecular subgroups [3]. Several therapeutics are being explored using this biomarker analysis. The TCGA endometrial cancer data expanded the knowledge about the role of different immunotherapeutic approaches based on molecular subtypes. Immune checkpoint inhibitors demonstrated distinct antitumor activities as monotherapy or in combination [4]. In microsatellite unstable (microsatellite instability-high) endometrial cancer, immune checkpoint inhibitors showed promising activity in recurrent settings. On the other hand, single immune checkpoint inhibitors showed underwhelming efficacy in microsatellite stable endometrial cancer but this improved using a combination approach.

Lurbinectedin (ZEPZELCA^™^) is an oncogenic transcription inhibitor that binds guanine-rich DNA sequences at gene promoters, evicts oncogenic transcription function and inhibits mRNA synthesis through ubiquitination and degradation of RNA polymerase II [5–7]. In a Basket, multicenter, open-label, phase 2 study (ClinicalTrials.gov identifier: NCT02454972), nine cohorts of patients with different difficult-to-treat tumor types received lurbinectedin to establish the proof of concept of anticancer activity for potential further clinical development. Based on the results in the small cell cancer (SCLC) cohort [8], approval of lurbinectedin was obtained first in the US [9] and in several other countries later (Canada, Australia, Switzerland, Singapore, South Korea, Ecuador, Mexico, Arab Emirates or Qatar). More recently, results of other cohort of this Basket trial have shown antitumor activity in relapsed Ewing sarcoma [10].

This report focuses on the outcomes of the endometrial cancer cohort. In addition, retrospective biomarker analysis based on TCGA and PromiSe molecular subtypes was explored. This cohort was evaluated because promising antitumor activity was previously found in a phase I study for a combination of doxorubicin plus lurbinectedin in patients with advanced endometrial cancer [11]. The overall response rate (ORR) of 42.1% was higher than the 14–16% reported for doxorubicin alone [12, 13], then suggesting a synergistic effect. Furthermore, another trial evaluating lurbinectedin plus paclitaxel showed an ORR of 27% in a small cohort of 11 patients with pretreated endometrial cancer [14]. In this study we report the activity and safety analysis of lurbinectedin monotherapy in addition to a translational exploratory analysis of endometrial molecular subtypes showing better PFS and OS in the *TP53* wild-type, low/absent p53 protein immunohistochemical (IHC) and No Specific Molecular Profile (NSMP) molecular subgroups.

## Methods

The study protocol was approved by the Independent Local Ethics Committee of each participating center and was conducted in accordance with the Declaration of Helsinki, Good Clinical Practice guidelines, and local regulations for clinical trials. Signed informed consent was obtained from all patients prior to any study-specific procedure. Additionally, patients were invited to participate in a translational study designed to identify molecular predictors of response or resistance to lurbinectedin, through an independent informed consent. The trial is registered at https://www.clinicaltrials.gov as NCT02454972.

### Patient selection

Seventy-three patients with endometrial cancer were treated at 19 investigational sites in Belgium (n = 3), France (n = 17), Germany (n = 2), Spain (n = 23), Switzerland (n = 2), the United Kingdom (n = 9), and the USA (n = 17). Eligibility criteria included patients ≥ 18 years old with pathologically proven diagnosis of endometrial carcinoma; pretreated with one prior adjuvant/advanced chemotherapy-containing line (including platinum or not); measurable disease as per the Response Criteria in Solid Tumors (RECIST) v.1.1 [15]; Eastern Cooperative Oncology Group performance status ≤ 2; and adequate major organ function. Patients were excluded if they had: previously received lurbinectedin or trabectedin; prior or concurrent malignant disease unless in complete remission for more than five years; known central nervous system involvement; concomitant unstable or serious medical condition, or impending need for radiotherapy.

### Lurbinectedin treatment

All patients were treated with lurbinectedin 3.2 mg/m^2^ administered as a 1-h intravenous (i.v.) infusion every three weeks (q3wk). All patients received antiemetic prophylaxis. Primary granulocyte colony-stimulating factors (G-CSFs) prophylaxis was not allowed. Treatment continued until disease progression, unacceptable toxicity, treatment delay > three weeks; more than two dose reductions; or patient refusal.

### Efficacy and safety assessments

The primary objective of this study was to assess the antitumor activity of lurbinectedin in terms of ORR, primary endpoint, assessed by the investigators. Radiological tumor evaluation was performed every six weeks (two cycles) until Cycle 6, and every nine weeks (three cycles) thereafter. Objective response was to be confirmed at least four weeks later. Secondary efficacy endpoints included disease control rate (ORR or stable disease), duration of response (DoR), progression-free survival (PFS), and OS.

Safety was evaluated in all patients who received at least one lurbinectedin infusion, complete or incomplete, by assessment of adverse events (AEs), clinical laboratory test results, physical examinations and vital signs. Laboratory tests were done weekly during Cycles 1 and 2, and on Day 1 of subsequent cycles. AEs were recorded and coded with the Medical Dictionary for Regulatory Activities (MedDRA), v.21.0. AEs and laboratory values were graded according to the National Cancer Institute-Common Toxicity Criteria for Adverse Events (NCI-CTCAE), v. 4.0. All patients were followed until recovery from any lurbinectedin-related AE.

### Translational study

Fifty of 73 treated patients (68.5%) had available archived formalin-fixed paraffin-embedded tumor samples and consented to participate in an optional translational study. In order to characterize patients’ tumors, a next generation sequencing (NGS) custom gene panel was performed, targeting 151 genes involved in cancer pathogenesis and DNA-repair (see Supplemental Methods). Data of sufficient quality was obtained for 42 patients. Moreover, to classify patients into four endometrial cancer molecular subgroups, two additional techniques were performed: microsatellite instability (MSI) status by fluorescent polymerase chain reaction (PCR) and IHC p53 protein staining (see Supplemental Methods). Analytically valid results were obtained for each technique for 47 and 50 samples respectively. Molecular sub-classification was obtained through an hierarchical algorithm [16, 17]: first, patients with pathogenic mutation in *POLE* gene exonuclease domain (POLE+ subgroup); second, MSI positives (dMMR/MSI subgroup); third, high staining abnormal p53 IHC (> 20% stained cells) and/or carriers of deleterious class 5 *TP53* mutations (p53 abnormal subgroup, p53 abn) (see Supplemental Methods) and, finally, the remaining were considered as “No Specific Molecular Profile” (NSMP subgroup).

### Statistical methods

Up to 50 evaluable patients were to be recruited to test the null hypothesis that 10% or less patients get a response (p ≤ 0.10) *versus* the alternative hypothesis that 25% or more patients get a response (p ≥ 0.25). The variance of the standardized test was based on the null hypothesis. The type I error (alpha) associated with this one-sided test is 0.025 and the type II error (beta) is 0.144; hence, statistical power is ~86%. With these assumptions, if the number of patients who achieve a confirmed response is ≥ 10, then this would allow the rejection of the null hypothesis.

Initially, 15 patients were to be included in a first stage. If one confirmed response occurred in the first 15 evaluable patients, recruitment had to continue up to 25 evaluable patients. Two of the first 15 patients had confirmed partial response (PR) to lurbinectedin treatment and, therefore, recruitment continued. Due to the signs of activity also observed in combination with doxorubicin [11], paclitaxel [14] or irinotecan [18], a protocol amendment was implemented to include 50 evaluable patients but, finally, because of the fast recruitment until the 50 evaluable patients were evaluated, 73 patients were enrolled and treated.

Descriptive statistics were used. Non-continuous variables are described in frequency tables using counts and percentages. Continuous variables are described by median, minimum and maximum. Binomial exact estimates and its 95% confidence interval (CI) were calculated for the evaluation of the main endpoint (ORR). The Kaplan-Meier method was used to analyze DoR, PFS and OS. For the translational sub-study analysis, the correlation between mutational status and OS or PFS was evaluated by a Cox regression analysis and Kaplan-Meier curves represented. SAS and R software were used to generate statistical outputs.

## Results

### Patient characteristics

Seventy-three patients were recruited and treated with lurbinectedin between 30 October 2016 and 19 April 2017. Cut-off for final analysis of all cohorts in this Basket study was 16 November 2020. Most patients were white (61.6%), with ECOG PS 0–1 (91.8%), and with a median age of 64 years (range, 32–80 years; 49.3% were ≥ 65 years old) (Table 1). The most common histological types were endometrioid (61.6%) and serous (27.4%). The median number of metastatic sites involved at baseline was 2 (range, 1–7), with 45.2% of patients having ≥ 3 disease sites. Lymph nodes (61.6%), lung (46.6%), peritoneum (45.2%) and liver (31.5%) were the most common disease sites. Sixty-two patients (84.9%) had previously undergone surgery. Prior radiotherapy had been administered to 39 patients (53.4%). The patients had received a median of one prior line of chemotherapy for advanced disease (range, 0–4 lines). The most common prior agents were carboplatin (95.9%) and paclitaxel (95.9%). ORR to last prior line was 37.0%.

**Table 1:** Baseline characteristics of the patients (n = 73)

	**n**	**%**
**Age: median (range), years**	64 (32–80)	
**Race**		
White	45	61.6
Other ^a^	22	30.1
Black of African American	5	6.8
Asian	1	1.4
**ECOG PS status**		
0–1	67	91.8
2	6	8.2
**BSA: median (range), m**^**2**^	1.8 (1.3–2.6)	
**Albumin: median (range), g/dL**	4.1 (2.7–4.7)	
**Stage at diagnosis**		
Early	23	31.5
Locally advanced	27	37.0
Metastatic	23	31.5
**Histological type**		
Endometrioid	45	61.6
Serous	20	27.4
Clear cell	4	5.5
Carcinosarcoma	3	4.1
Other ^b^	1	1.4
**No. of sites at baseline: median (range)**	2 (1–7)	
≥ 3 sites	33	45.2
**Most common sites of disease at baseline**		
Lymph nodes	45	61.6
Lung	34	46.6
Peritoneum	33	45.2
Liver	23	31.5
Primary site	15	20.5
Soft tissue	13	17.8
Bone	9	12.3
Pleura	6	8.2
**Bulky disease (one lesion > 50 mm)**	16	21.9
**Prior therapy**		
Surgery	62	84.9
Radiotherapy	39	53.4
**No. of prior advanced chemotherapy lines: median (range)**	1 (0–4)	
Most common prior agents		
Platinum compounds	72	98.6
Taxanes	70	95.9
Anthracyclines	4	5.5
Bevacizumab	4	5.5
Immunotherapy (Pembrolizumab)	1	1.4
mTOR inhibitors (Everolimus)	1	1.4
PARPi (Olaparib)	1	1.4
**Prior endocrine therapy**		
Aromatase inhibitors	8	11.9
Progestogens	8	11.0
Gonadotropin-releasing hormone analogues	1	1.4
Tamoxifen	1	1.4
**Best response to last therapy**		
CR	7	9.6
PR	20	27.4
SD	13	17.8
PD	15	20.5
Unknown/not available	18	24.7

Data shown are n (%) of patients except for median (range)

*BSA* body surface area, *CR* complete response, *ECOG PS* Eastern Cooperative Oncology Group Performance Status, *PARP* poly (ADP-ribose) polymerase, *PD* disease progression, *PR* partial response, *SD* stable disease

^a^Two patients were Hispanic or Latino. Furthermore, patients recruited in France and Belgium had not race available because of specific ethical requirements in these countries

^b^Endometrial stromal sarcoma (epithelioid)

### Lurbinectedin treatment

A total of 378 cycles were administered to the 73 treated patients. The median number of cycles per patient was 4 (range, 1–22 cycles), with 31.5% of patients having received ≥ 6 cycles. The median relative dose intensity was 97.7% (range, 64.9–102.9%). Twenty patients had treatment-related dose delays, being hematological toxicity the most common reason: grade 2–4 neutropenia in 13 patients and grade 3 anemia in two patients. Lurbinectedin dose was reduced due to treatment-related reasons in 6.9% of cycles in 20 patients, being hematological toxicity the most common cause: grade 2-4 neutropenia in eight patients and eight cycles; grade 3/4 febrile neutropenia in two patients and two cycles; and grade 3 leukopenia in one patient and one cycle. Of note, the protocol stated that in case of grade 4 neutropenia, lurbinectedin dose had to be reduced instead of continuing at the same dose with granulocyte colony-stimulating factor (G-CSF) prophylaxis.

### Efficacy results

Seventy-one patients were evaluable for efficacy (Table 2). Two patients were not evaluable due to patient refusal prior to the first disease measurement, and death because of grade 5 septic shock considered unrelated to the study treatment in Cycle 1.

**Table 2:** Efficacy results with lurbinectedin treatment in patients with pretreated endometrial cancer (n = 71 evaluable patients)

**RECIST responses (n, %)**	
CR	2 (2.9%)
PR	6 (8.5%)
SD	29 (40.8%)
SD ≥ 4 months	17 (23.9%)
PD	30 (42.3%)
Not evaluable	4 (5.6%)
ORR, % (95%CI)	11.3% (5.0–21.0%)
Clinical benefit rate (CR + PR + SD ≥ 4 months), % (95%CI)	35.2% (24.2–47.5%)
Disease control rate ^b^ (CR + PR + SD), % (95%CI)	52.1% (39.9–64.1%)

**Duration of Response (DoR)**	
Median, months (95%CI)	9.2 (3.4–18.0)
DoR at 6 months, % (95%CI)	71.4% (38.0–104.9%)

**Progression-free survival (PFS)**	
Median, months (95%CI)	2.6 (1.4–4.0)
PFS at 6 months, % (95%CI)	29.0% (18.2–39.8%)

**Overall survival (OS)**	
Median, months (95%CI)	9.3 (6.1–12.8)
OS at 12 months, % (95%CI)	45.8% (33.8–75.9%)

*CI* confidence interval, *CR* complete response, *DoR* duration of response, *OS* overall survival, *PD* disease progression, *PFS* progression-free survival, *PR* partial response, *RECIST* Response Evaluation Criteria in Solid Tumors, *SD* stable disease

**Fig. 1:**
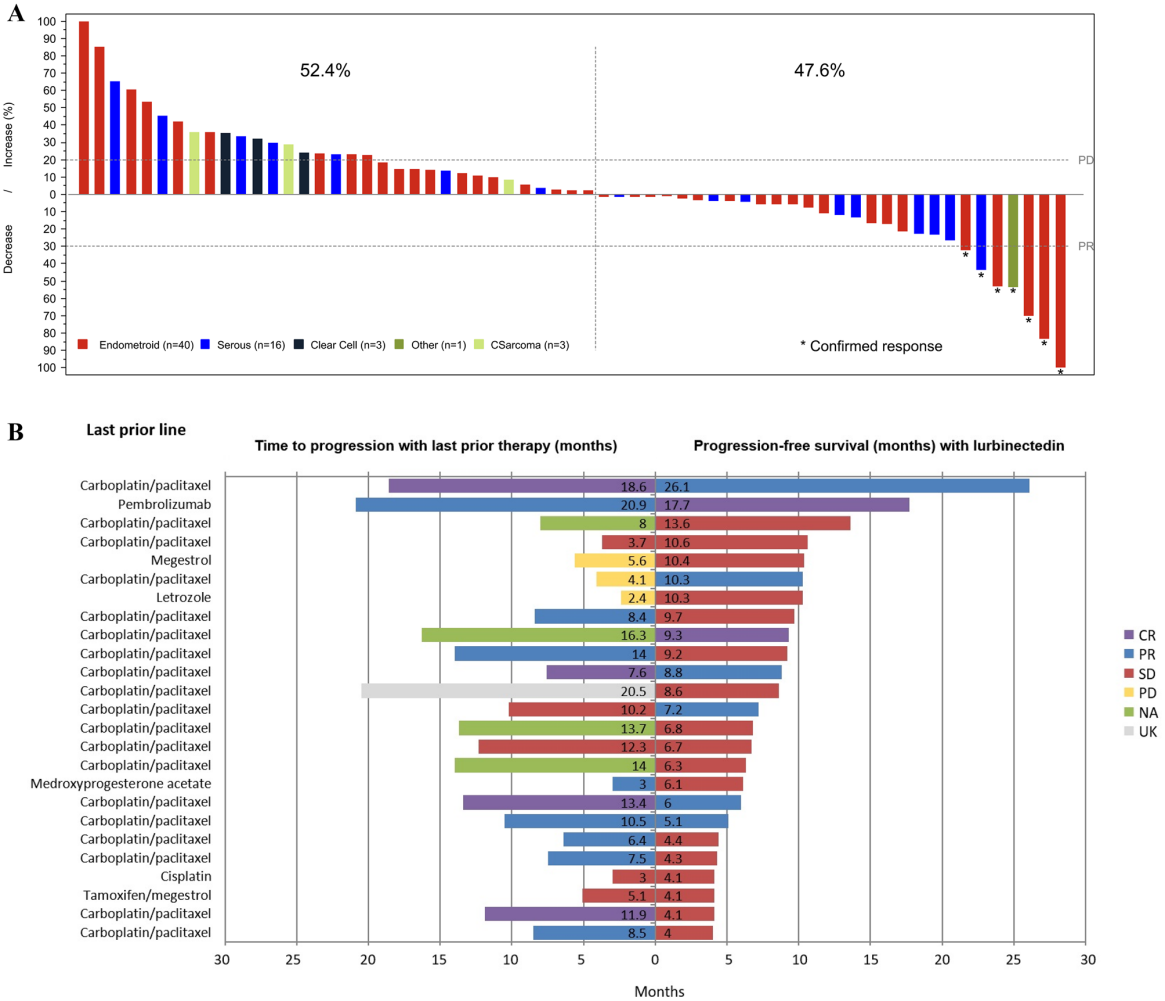
**A** Waterfall plot showing maximum variation of target lesions size with lurbinectedin in patients with pretreated endometrial cancer. **B** Time to progression with last prior therapy (months) *versus* progression-free survival (months) with lurbinectedin in patients with endometrial cancer and clinical benefit (complete response, partial response or stable disease ≥ 4 months). Abbreviations: CR, complete response; NA, not available; PD, disease progression; PR, partial response; SD, stable disease; UK, unknown

Confirmed complete response (CR) was reported in two patients (2.9%) and partial response (PR) in six patients (8.5%). Stable disease (SD) was observed in 29 patients (40.8%), with 17 of them (23.9%) reaching SD ≥ 4 months. Therefore, ORR was 11.3% (95%CI, 5.0–21.0%). Overall, 47.6% of patients had reduction in target lesions during the treatment period (Fig. 1A). Objective responses were observed in the two most common histological subtypes: endometrioid (five responses) and serous (two responses). The other response was reported in a patient with endometrial stromal sarcoma (epithelioid). The median of prior lines in responder patients was one (range, 1–4) (Supplemental Table 1).

Median DoR was 9.2 months (95%CI, 3.4–18.0 months). Clinical benefit rate (CR + PR + SD ≥ 4 months) and disease control rate (CR + PR + SD) were 35.2% (95%CI, 24.2–47.5%) and 52.1% (95%CI, 39.9–64.1%), respectively (Table 2). Median duration for clinical benefit rate and disease control rate was 7.1 months and 5.6 months, respectively. Time to progression with last prior therapy *versus* PFS with lurbinectedin is shown in Fig. 1B.

Median PFS was 2.6 months (95%CI, 1.4–4.0 months) and PFS rate at 6 months was 29.0% (95%CI, 18.2–39.8%). With a median follow-up of 28.9 months and a censoring rate of 19.8%, median OS was 9.3 months (95%CI, 6.1–12.8 months) (Table 2).

Thirty-five patients (47.9%) received further antitumor medical therapy, nine patients (12.3%) received further radiotherapy and two patients (2.7%) underwent further surgery after lurbinectedin. The most common agents subsequently received were paclitaxel (n = 14; 19.2%) and carboplatin (n = 13; 17.8%).

### Translational study results

**Fig. 2:**
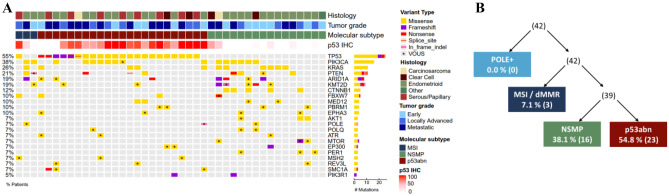
**A** Oncoplot showing mutational profile on every patient sample, together with histology, tumor grade, p53 immunohistochemistry and molecular subtype classification. **B** Molecular classification algorithm for endometrial cancer patients included in this cohort. Abbreviations: IHC, immunohistochemistry; VOUS, variants of unknown significance; MSI/dMMR, “microsatellite instable/mismatch repair deficient” molecular subgroup; NSMP, “No Specific Molecular Profile” molecular subgroup; p53abn, “p53 abnormal” molecular subgroup including abnormal p53 IHC and nonsense/non-funtional *TP53* mutants

In the context of a retrospective translational study, an NGS panel was performed to identify molecular tumor biomarkers that might influence the clinical response to lurbinectedin. The mutational landscape observed was typical for an endometrial cancer cohort [2]: *TP53* mutated (54.8% of tumors), *PIK3CA* (38.0%), *PTEN* (21.4%), *KRAS* (26.2%), and *KMT2D* and *ARID1A* (19.0% each) (Fig. 2A). The most remarkable results are shorter median PFS in patients with *PIK3CA* mutation positive tumors: 2.0 *vs.* 4.0 months in the wild-type group (p = 0.0059), and shorter median OS in patients with *TP53* pathogenic mutation: 6.6 *vs.* 16.1 months in the wild-type group (p = 0.0020) (Supplemental Table 2 and Supplemental Fig. 1A-D).

In addition to the traditional classification, based on staging and histology, last guidelines recommend to incorporate tumor biomarkers to allow endometrial cancer subgroup classification in four molecular subtypes: p53 abnormal, dMMR/MSI+, POLE-mutated, and “no specific molecular profile” (NSMP) [16, 17]. To classify patient’s tumors, NGS characterization was complemented with evaluation of MSI status and p53 protein IHC staining (see Supplemental Methods). None of the samples was carrier of any described or likely pathogenic *POLE* variant; three samples (7.1%) were MSI positive, 23 samples (54.8%) showed p53/*TP53* inactivation and 16 samples (38.1%) were classified as NSMP (Fig. 2B). Consistently with observations on *TP53* pathogenic mutation, as under normal conditions wild-type p53 protein is rapidly degraded and inactive p53 accumulates [19], patients with high p53 IHC staining showed a numerically shorter mean PFS than p53 low/absent normal tumors (1.7 months *vs.* 2.7 months, p = 0.3309) and a shorter median OS (8.2 months *vs.* 12.8 months, p = 0.0345) (Supplemental Table 3 and Supplemental Fig. 1E-F).

Molecular subtypes showed differences in PFS, with a difference at 6 months of 19.2% between p53abn and NSMP molecular subgroups, 23.7% (95%CI, 5.8%-41.7%) and 42.9% (95%CI, 18.2%-67.5%) respectively (Supplemental Table 4). Overall survival was significantly shorter in p53 abnormal group with a median of 6.6 months (95%CI, 3.1–12.1) compared to the NSMP group, with a median of 16.1 months (95%CI, 5.3–26.6) (Supplemental Table 4 and Supplemental Fig. 1G-H). No significant differences were seen on MSI molecular subgroup (data not shown).

### Safety results

All 73 treated patients were evaluable for safety (Table 3). The most common treatment-related adverse events were fatigue (54.8% of patients), gastrointestinal disorders (nausea, 50.7%, vomiting, 26.0%, and constipation, 19.2%), and metabolism and nutrition disorders (mainly decreased appetite, 17.8%). These adverse events were mostly grade 1/2. The most common treatment-related grade 3/4 AEs and laboratory abnormalities regardless of relationship were hematological disorders including anemia (27.4%), leukopenia (32.9%) and neutropenia (43.8%; grade 4, 19.2%; febrile neutropenia, 4.1%); fatigue (4.1%), nausea (2.7%), diarrhea (2.7%), and increased liver function tests, including increased transaminases (ALT, 4.2%; AST, 1.4%), alkaline phosphatase (5.6%) and GGT (19.2%). Eleven patients (15.1%) received G-CSFs secondary prophylaxis or therapeutic for neutropenia.

**Table:** Most common laboratory abnormalities and treatment-related adverse events (≥ 10% of patients or grade ≥ 3) in patients with pretreated endometrial cancer (n = 73)

	**NCI-CTCAE grade**									
	**Grade 1–2**		**Grade 3**		**Grade 4**		**Grade 5**		**Total**	
	**n**	**%**	**n**	**%**	**n**	**%**	**n**	**%**	**n**	**%**
**Hematological abnormalities (regardless of relationship)**										
Anemia	46	63.0	19	26.0	1	1.4	-	-	66	90.4
Leukopenia	34	46.6	15	20.5	9	12.3	-	-	58	79.5
Neutropenia	19	26.0	18	24.7	14	19.2	-	-	51	69.9
Thrombocytopenia	30	41.1	3	4.1	2	2.7	-	-	35	47.9

**Biochemical abnormalities (regardless of relationship)**^**a**^										
Creatinine increased ^b^	63	86.3	2	2.7	-	-	-	-	65	89.0
GGT increased	37	50.7	14	19.2	1	1.4	-	-	52	71.2
ALT increased	43	58.9	3	4.2	-	-	-	-	46	63.9
AST increased	38	52.1	1	1.4	-	-	-	-	39	54.2
AP increased	32	43.8	4	5.6	-	-	-	-	36	50.0
Total bilirubin increased	9	12.7	2	2.8	-	-	-	-	11	15.5
CPK increased	9	12.7	-	-	-	-	-	-	9	12.7

**Treatment-related adverse events**										
Fatigue	37	50.7	3	4.1	-	-	-	-	40	54.8
Nausea	35	47.9	2	2.7	-	-	-	-	37	50.7
Vomiting	18	23.8	1	1.4	-	-	-	-	19	26.0
Constipation	14	19.2	-	-	-	-	-	-	14	19.2
Decreased appetite	13	17.8	-	-	-	-	-	-	13	17.8
Diarrhea	9	12.3	2	2.7	-	-	-	-	11	15.1
Peripheral neuropathy	4	5.5	1	1.4	-	-	-	-	5	6.8
Abdominal pain	3	4.1	1	1.4			-	-	4	5.5
Febrile neutropenia	-	-	2	2.7	1	1.4	-	-	3	4.1
Peripheral edema	2	2.7	1	1.4	-	-	-	-	3	4.1
Dehydration	1	1.4	1	1.4	-	-	-	-	2	2.8
Sepsis	-	-	-	-	-	-	1	1.4 ^c^	1	1.4
Ataxia	-	-	1	1.4	-	-	-	-	1	1.4
Vertigo	-	-	1	1.4	-	-	-	-	1	1.4

*AP* alkaline phosphatase, *ALT* alanine aminotransferase, *AST* aspartate aminotransferase, *CPK* creatine phosphokinase, *GGT* gamma glutamyltransferase, *NCI-CTCAE* National Cancer Institute Common Terminology Criteria for Adverse Events v.4

^a^Based on patients with laboratory data available (ranging from 71 to 73 depending on the parameter)

^b^Version 4.0 of NCI-CTCAE grades creatinine increases from baseline, even if creatinine values remain normal

^c^One patient died due to grade 5 sepsis infection after two cycles

One patient died due to treatment-related grade 5 sepsis infection after two cycles (Table 3). This case was associated with severe neutropenia. During hospitalization, blood culture was positive for *Klebsiella*, *Escherichia coli*, *Streptococcus viridans* and *Streptococcus* and CT-scan showed disease progression that was later confirmed in the autopsy.

Most patients (n = 59; 80.8%) discontinued the study treatment due to disease progression. With respect to the other 14 patients, five (6.8%) died while on treatment (three due to disease progression, one due to grade 5 septic shock unrelated to treatment, and one due to treatment-related grade 5 sepsis, above explained); five (6.8%) refused to continue treatment; one (1.4%) discontinued lurbinectedin therapy due to a treatment-related adverse event: persistence of peripheral neuropathy (grade 2 was present at baseline and worsened to grade 3); and the other three patients discontinued lurbinectedin due to adverse events unrelated to the study treatment (n = 1) or because of investigator decision based on benefit-risk balance (n = 2).

## Discussion

This cohort from a phase 2 exploratory Basket study included 73 patients with pretreated endometrial cancer who received therapy with single-agent lurbinectedin. ORR according to RECIST v.1.1 was 11.3% (95%CI, 5.0–21.0%). Responses were mostly observed in patients with endometrioid tumors and the median of prior lines was one. These results (eight objective responses) were lower than the threshold of ≥ 10 confirmed responses established in the statistical hypothesis for this endometrial carcinoma cohort. However, although the cohort did not meet the planned hypothesis, hints of antitumor activity were observed, with two patients achieving complete response and six patients with partial responses in a quite heterogeneous cohort that included patients with different number of prior lines administered (up to four previous lines), different histology subtypes (e.g., carcinosarcoma, endometrial stromal sarcoma), and not characterized molecularly at study entry according to current guidelines [20]. Of note, median duration of response was prolonged (9.2 months). This duration of response was similar to that found for physician’s choice following platinum-based therapy in patients with advanced endometrial cancer in a recent phase 3 study [21].

Studies in small cohorts of patients of lurbinectedin in combination with other drugs have shown to increase the single-agent activity in pretreated endometrial cancer. For instance, in combination with doxorubicin (ORR = 42% and median DoR = 7.5 months) [11], paclitaxel (ORR = 27% and median DoR = 6.1 months) [14], or irinotecan (ORR = 30%; median DoR not available) [18].

Retrospective tumor molecular and genomic profiling have shown different lurbinectedin response depending on the presence of mutations on particular genes, protein levels and specific molecular subtypes, with better PFS and OS in the *TP53* wild-type, low/absent p53 protein IHC and NSMP molecular subgroup. These biomarkers/molecular classification might help to identify those patients who could get more benefit from lurbinectedin alone or in combination. However, as molecular subgroups are known to have a prognostic value [17] and no control arm was included in this trial, these results should be taken with caution and considered merely as hypothesis-generating. In any case, our results exemplified how molecular testing/classification should be incorporated in endometrial cancer clinical trials in the same extension as it is nowadays recommended to be included in the clinical management of endometrial cancer [16, 17].

Lurbinectedin administered at 3.2 mg/m^2^ as a 1-h i.v. q3wk infusion in patients with pretreated endometrial carcinoma demonstrates a predictable and manageable safety profile, with the main toxicity being reversible myelosuppression, fatigue and nausea/vomiting. Overall, the safety profile reported for lurbinectedin in this cohort of patients agrees with the results observed previously in patients with other solid tumors such as breast cancer [22], Ewing sarcoma [10] neuroendocrine tumors [23], ovarian cancer [24, 25], or SCLC [8].

In conclusion, the current efficacy results suggest that antitumor activity of lurbinectedin could be improved in patients with pretreated endometrial cancer when administered in combination with other agents and in populations with previous molecular classification. Immunotherapy added to chemotherapy has shown promising results in the first-line treatment of endometrial cancer [26–30]. Immunotherapy currently is placed in the second-line setting in advanced treatment of endometrial cancer, as single agent in deficient mismatch repair (dMMR), or in combination (e.g., pembrolizumab-lenvatinib) in proficient mismatch repair (pMMR). The evaluation of lurbinectedin combined with an immune checkpoint inhibitor in pMMR is warranted.

### Supplementary Information

Below is the link to the electronic supplementary material.

Supplementary file1 (DOCX 230 KB)

## Data Availability

Individual participant data are not publicly available since this requirement was not anticipated in the study protocol considering that this trial started patient enrolment in 2015. Clinical trial summary results were placed in the European Clinical Trials Database (EudraCT; https://eudract.ema.europa.eu; study 2014-003773-42) and ClinicalTrials.gov (Identifier: NCT02454972).
