# Supplementary material for: Lurbinectedin in patients with pretreated endometrial cancer: results from a phase 2 basket clinical trial and exploratory translational study
Source: Invest New Drugs. 2023 Aug 9;41(5):677–87. doi: 10.1007/s10637-023-01383-2 (PMC10560193; doi:10.1007/s10637-023-01383-2)
Supplement: Supplementary file 1 — Supplementary file1 (DOCX 230 KB) [file 10637_2023_1383_MOESM1_ESM.docx]

SUPPLEMENTAL MATERIAL

**Supplemental Methods**

A minimum of 70% tumor cells was required. Genomic DNA was extracted from archived formalin-fixed paraffin-embedded (FFPE) tumors.

*Next Generation Sequencing (NGS) gene panel*

A custom Next Generation Sequencing (NGS) gene panel optimized for somatic determinations was designed using Ampliseq Designer tool (ThermoFisher Scientific), targeting the coding sequence of 151 genes involved in cancer pathogenesis and in DNA-repair: *AKT1, ALKBH2, ALKBH3, APC, APEX1, APTX,ARID1A, ARID2, ARID5B, ASXL1, ATM, ATR, ATRX, BAP1, BLM, BRAF, BRCA1, BRCA2, BRIP1, CCND1, CDH1, CDK12, CDKN2A, CDKN2C, CEBPA, CHEK1, CHEK2, CREBBP, CTNNB1, DDB1, DDB2, DMC1, DNMT3A, EGFR, EP300, EPHA3, EPHB6, ERBB2, ERBB3, ERBB4, ERCC1, ERCC2, ERCC3, ERCC4, ERCC5, ERCC6, EXO1, EZH2, FANCA, FANCC, FANCD2, FANCE, FANCF, FANCG, FANCM, FBXW7, FEN1, FGFR2, FGFR3, FLT3, GATA3, GEN1, HRAS, IDH1, IDH2, KDM5C, KDM6A, KMT2A, KMT2C, KMT2D, KEAP1, KIT, KRAS, LIFR, LIG1, LIG3, LIG4, MAP2K4, MED1, MED12, MLH1, MLH3, MRE11A, MSH2, MSH6, MTOR, MUTYH, NBN, NEIL1, NEIL3, NF1, NFE2L2, NHEJ1, NOTCH1, NPM1, NRAS, NSD1, OGG1, PALB2, PARP1, PBRM1, PER1, PIK3CA, PIK3R1, PMS1, PMS2, POLD1, POLE, POLQ, PRKDC, PTEN, RAD21, RAD50, RAD51, RAD51B, RAD51C, RAD51D, RAD54B, RB1, RBBP8, RECQL4, RECQL5, REV3L, RUNX1, SETBP1, SETD2, SF3B1, SHPRH, SMAD2, SMAD4, SMC1A, SMC3, SMUG1, STAG2, STK11, TET1, TET2, TGFBR2, TOPBP1, TP53, VHL, WRN, WT1, XPA, XPC, XRCC1, XRCC2, XRCC3, XRCC4, XRCC5* and *XRCC6*.

Libraries were sequenced on an Ion S5™ System Sequencer (ThermoFisher Scientific) following manufacturer recommendations. Primary and secondary processing were performed using Torrent Suite/Ion Reporter softwares (ThermoFisher Scientific). An in-house bioinformatics pipeline was designed to filter-out sequencing artifacts, to annotate variants using public databases such as gnomAD, CancerHotspots, Civic, Cancer Genome Interpreter or ClinVar, or to infer putative loss-of-function in tumor suppressor genes. Tumor genetic variants were classified as benign, likely-benign, variants of unknown significance (VOUS), likely oncogenic and oncogenic. Only missense, nonsense and indels in coding and proximal splice regions, excluding likely benign and benign variants, were considered for further analysis.

*Microsatellite instability (MSI) status*

Microsatellite instability (MSI) status was studied in tumor-only DNA samples using the fluorescence PCR kit MSI Analysis System (Promega) following the manufacturer’s instructions. Microsatellite BAT-25, BAT-26, MONO-27, NR-21 and NR-24 markers were resolved by capillary electrophoresis.

*Immunohistochemical (IHC) p53 protein staining*

Immunohistochemical (IHC) p53 protein staining was performed on FFPE slides using monoclonal DO-7 mouse anti-human p53 antibody. Results were shown as the percentage of p53 staining positive cells. Due to the very short half-life of wild-type p53, non-detectable p53 IHC staining is interpreted as functional p53. Non-functional missense *TP53* mutants avoids signaling leading to p53 degradation, thus accumulating and being detectable by IHC. Biallelic truncating and/or loss-of-heterozygosity *TP53* mutations usually lead to lack of p53 staining ([1](#_ENREF_1)). Expression levels higher than 20% were considered as positive.

Supplemental Table 1. Characteristics of endometrial cancer patients with objective response to lurbinectedin.

| **Age/ ECOG PS** | **Histology Type** | **Prior Lines** | **Last Therapy / Best Response** | **Cycles Received** | **Best Response** | **Location sites** | **Sum of lesions at baseline (mm)** | **Maximum reduction in lesions (%)** | **DoR (months)** | **PFS (months)** | **OS (months)** |
| --- | --- | --- | --- | --- | --- | --- | --- | --- | --- | --- | --- |
| 75/0 | Endometrioid | 4 | Pembrolizumab / PR | 22 | CR | Lymph node | 18 | 100% | 16.6 | 17.7 | 34.4+ |
| 64/0 | Serous | 1 | Carboplatin/paclitaxel / NA | 12 | CR | ^a^ | - | - | 7.6+ | 9.3+ | 9.7+ |
| 66/0 | Endometrioid | 1 | Carboplatin/paclitaxel / SD | 10 | PR | Lung/peritoneal | 30 | 73% | 4.3 | 7.2 | 26.6 |
| 61/1 | Endometrial stromal sarcoma (epithelioid). | 2 | Carboplatin/paclitaxel / PD | 15 | PR | Liver/left flank | 133 | 53% | 9.2 | 10.3 | 20.8 |
| 72/1 | Serous | 1 | Carboplatin/paclitaxel / CR | 12 | PR | Lymph node | 16 | 44% | 7.5 | 8.8 | 14.6 |
| 69/0 | Endometrioid | 1 | Carboplatin/paclitaxel / CR | 9 | PR | Lymph node/peritoneum | 32 | 53% | 3.4 | 6.0 | 19.0 |
| 75/1 | Endometrioid | 1 | Carboplatin/paclitaxel / PR | 9 | PR | Lung | 25 | 32% | 2.0+ | 5.1+ | 28.6+ |
| 73/1 | Endometrioid | 2 | Carboplatin/paclitaxel / CR | 4 | PR | Lymph node | 18 | 83% | 18.0 | 26.1 | 26.1 |
| ^a^ No target lesions were present at baseline. Response was based on non-target lesions in lymph nodes (retrocaval and parailiacal).  Abbreviations: CR, complete response; DoR, duration of response; ECOG, Eastern Cooperative Oncology group; NA, not available; OS, overall survival; PD, disease progression; PFS, progression-free survival; PR, partial response, PS, performance status; SD; stable disease. | | | | | | | | | | | |

**Supplemental Table 2.** Progression-free survival and overall survival of endometrial cancer patients treated with lurbinectedin comparing mutated and wild-type top 5 mutated genes.

|  | **Mutated** | **Wild-type** |  |
| --- | --- | --- | --- |
| **Gene** | **Median (95%CI) (months)** | **Median (95%CI) (months)** | **Log-rank test p-value** |
| **Progression-free survival (PFS)** | | | |
| ***ARID1A*** | 2.0 (1.2-4.1) | 2.8 (1.4-6.0) | 0.1387 |
| ***KRAS*** | 4.0 (1.2-7.2) | 2.7 (1.4-4.2) | 0.7223 |
| ***PIK3CA*** | 2.0 (1.2-4.0) | 4.0 (1.4-7.2) | 0.0059 |
| ***PTEN*** | 2.7 (0.8-6.1) | 2.8 (1.4-4.3) | 0.5870 |
| ***TP53*** | 2.8 (1.3-4.2) | 2.7 (1.3-7.2) | 0.4615 |
| **Overall survival (OS)** | | | |
| ***ARID1A*** | 6.3 (2.6-12.5) | 12.1 (4.5-15.1) | 0.3433 |
| ***KRAS*** | 8.2 (3.2-nr) | 12.1 (3.3-13.1) | 0.2208 |
| ***PIK3CA*** | 9.3 (2.4-13.1) | 12.1 (5.3-16.6) | 0.0983 |
| ***PTEN*** | 13.1 (1.0-16.6) | 9.9 (3.3-12.5) | 0.4101 |
| ***TP53*** | 6.6 (3.1-12.1) | 16.1 (5.3-26.6) | 0.0020 |
| CI, confidence interval. | | | |

**Supplemental Table 3.** Progression-free survival and overall survival of endometrial cancer patients treated with lurbinectedin by p53 IHC staining.

|  | **<20%**  **(n=21)** | **>20%**  **(n=29)** | **Log-rank test**  **p-value** |
| --- | --- | --- | --- |
| **Progression-free survival (PFS)** | | | |
| **Events** | 20 (95.2%) | 27 (93.1%) |  |
| **Censored** | 1 (4.8%) | 2 (6.9%) |  |
| **Median, months (95%CI)** | 2.7 (1.4-7.2) | 1.7 (1.3-4.0) | 0.3309 |
| **PFS at 4 months, % (95%CI)** | 47.6% (26.3-69.0) | 33.6% (16.2-51.1) |  |
| **PFS at 6 months, % (95%CI)** | 42.3% (21.0-63.7) | 18.7% (4.1-33.3) |  |
| **Overall survival (OS)** | | | |
| **Events** | 16 (76.2%) | 25 (86.2%) |  |
| **Censored** | 5 (23.8%) | 4 (13.8%) |  |
| **Median, months (95%CI)** | 12.8 (6.6-20.9) | 8.2 (3.2-12.1) | 0.0345 |
| **OS at 6 months, % (95%CI)** | 79.9% (62.2-97.6) | 57.4% (39.1-75.7) |  |
| **OS at 12 months, % (95%CI)** | 58.6% (36.5-80.7) | 39.1% (20.9-57.3) |  |

**Supplemental Table 4.** Progression-free survival and overall survival of endometrial cancer patients treated with lurbinectedin by molecular subtype.

|  | **MSI**  **(n=3)** | **p53abn**  **(n=23)** | **NSMP**  **(n=16)** | **Log-rank test**  **p-value** |
| --- | --- | --- | --- | --- |
| **Progression-free survival (PFS)** | | | | |
| **Events** | 3 (100%) | 21 (91.3%) | 15 (93.8%) |  |
| **Censored** | 0 (0%) | 2 (8.7%) | 1 (6.3%) |  |
| **Median, months (95%CI)** | 2.5 (1.5-4.0) | 2.8 (1.3-4.2) | 3.4 (1.3-8.6) | 0.3764 |
| **PFS at 4 months, % (95%CI)** | 33.3% (0-86.7) | 37.9% (17.7-58.2) | 50.0% (25.5-74.5) |  |
| **PFS at 6 months, % (95%CI)** | 0% | 23.7% (5.8-41.7) | 42.9% (18.2-67.5) |  |
| **Overall survival (OS)** | | | | |
| **Events** | 2 (66.7%) | 22 (95.7%) | 11 (68.8%) |  |
| **Censored** | 1 (33.3%) | 1 (4.3%) | 5 (31.3%) |  |
| **Median, months (95%CI)** | 13.1 (3.3-nr) | 6.6 (2.9-12.1) | 16.1 (5.3-26.6) | 0.0069 |
| **OS at 6 months, % (95%CI)** | 66.7% (13.3-100.0) | 56.5% (36.3-76.8) | 78.7% (57.0-100.0) |  |
| **OS at 12 months, % (95%CI)** | 66.7% (13.3-100.0) | 39.1% (19.2-59.1) | 62.9% (36.9-89.0) |  |

**Supplemental Figure 1.** Kaplan-Meier curves for PFS (A, C, E, G) and OS (B, D, F, H) comparing *PI3KCA* mutated *vs.* wild type (A-B), *TP53* mutated *vs.* wild type (C-D), p53 >20% positive cells *vs.* p53 <20% positive cells normal IHC (E-F) and NSMP *vs.* p53abn molecular subgroups (G-H).

**
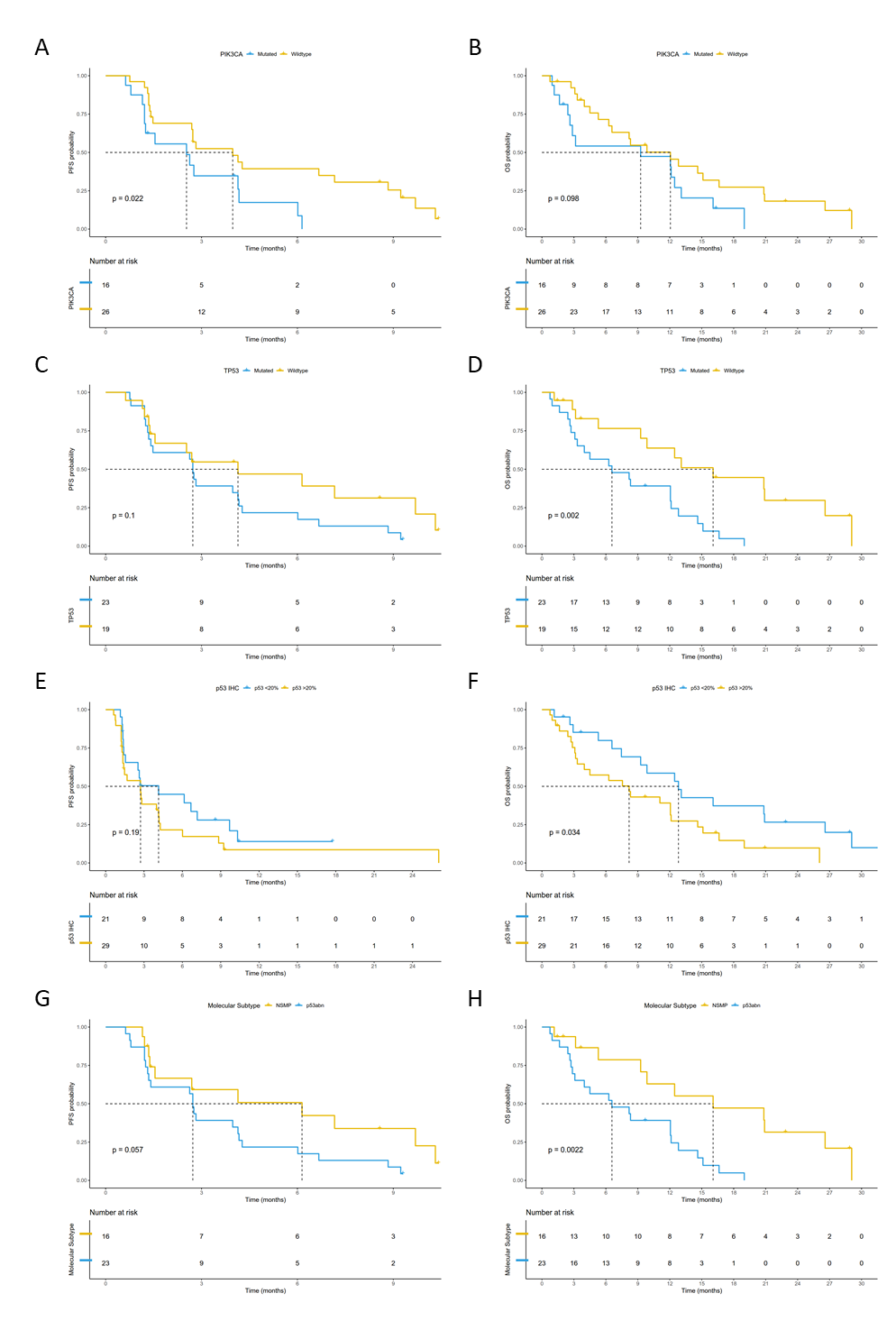
**

**References**

1. Kobel M, Ronnett BM, Singh N, Soslow RA, Gilks CB, McCluggage WG. Interpretation of P53 Immunohistochemistry in Endometrial Carcinomas: Toward Increased Reproducibility. Int J Gynecol Pathol 2019;38 Suppl 1:S123-S31
